# The Cellular Localization of Human Cytomegalovirus Glycoprotein Expression Greatly Influences the Frequency and Functional Phenotype of Specific CD4^+^ T Cell Responses

**DOI:** 10.4049/jimmunol.1500696

**Published:** 2015-09-11

**Authors:** Annette Pachnio, Jianmin Zuo, Gordon B. Ryan, Jusnara Begum, Paul A. H. Moss

**Affiliations:** * School of Cancer Sciences, College of Medicine and Dentistry, University of Birmingham, Birmingham B15 2TT, United Kingdom; and; † University Hospitals Birmingham National Health Service Foundation Trust, Birmingham B15 2TH, United Kingdom

## Abstract

CMV infection is a significant cause of morbidity and mortality in immunocompromised individuals, and the development of a vaccine is of high priority. Glycoprotein B (gB) is a leading vaccine candidate but the glycoprotein H (gH) pentameric complex is now recognized as the major target for neutralizing Abs. However, little is known about the T cell immune response against gH and glycoprotein L (gL) and this is likely to be an important attribute for vaccine immunogenicity. In this study, we examine and contrast the magnitude and phenotype of the T cell immune response against gB, gH, and gL within healthy donors. gB-specific CD4^+^ T cells were found in 95% of donors, and 29 epitopes were defined with gB-specific response sizes ranging from 0.02 to 2.88% of the CD4^+^ T cell pool. In contrast, only 20% of donors exhibited a T cell response against gH or gL. Additionally, gB-specific CD4^+^ T cells exhibited a more cytotoxic phenotype, with high levels of granzyme B expression. Glycoproteins were effectively presented following delivery to APCs but only gB-derived epitopes were presented following endogenous synthesis. gB expression was observed exclusively within vesicular structures colocalizing with HLA-DM whereas gH was distributed evenly throughout the cytoplasm. Grafting of the C-terminal domain from gB onto gH could not transfer this pattern of presentation. These results reveal that gB is a uniquely immunogenic CMV glycoprotein and this is likely to reflect its unique pattern of endogenous Ag presentation. Consideration may be required toward mechanisms that boost cellular immunity to gH and gL within future subunit vaccines.

## Introduction

Cytomegalovirus can cause severe disease in the setting of congenital infection or immune suppression, and development of a CMV vaccine has been given high priority by the Institute of Medicine (1–6). Such a vaccine would have two main aims: first, the induction of neutralizing Abs to prevent vertical transmission as a means to prevent congenital CMV infection; second, the induction or boosting of T cell immunity in individuals that already carry the virus may improve the virus–host balance within patients such as those receiving solid organ or stem cell transplants. This latter ambition is supported by substantial evidence underpinning the role of virus-specific T cells in controlling viral replication, especially in the setting of allogeneic transplantation (7–11). A particular role for CD4^+^ T cells has also been shown in reducing viral transmission at time of primary infection during pregnancy (12).

The principal target protein to date, and the most advanced in terms of vaccine development, has been glycoprotein B (gB), one of the most abundant proteins within the viral envelope and important for viral entry (13, 14). Abs against gB can prevent viral infection of fibroblast target cells (15, 16), and a number of vaccines have been developed, including adjuvanted gB protein, DNA vaccines encoding gB and pp65, and alphavirus replicon particles expressing gB, pp65, and IE-1 (17–20). Initial studies demonstrated a 50% efficacy in protecting women against primary infection and a reduction in the duration of viremia and requirement for antiviral treatment following solid organ transplantation in CMV-seronegative recipients. However, recent results from multicenter studies suggest somewhat less efficacy in relation to prevention of primary infection (21), and there is a considerable need to improve the efficacy of next-generation vaccines. Importantly, recent investigations have shown that the gH pentameric complex, containing glycoprotein H (gH), glycoprotein L (gL), UL128, UL130, and UL131A, is essential for viral entry into epithelial and endothelial cells (22), which represent principal target cells of CMV infection in vivo. Furthermore, most neutralizing Abs are directed against this complex rather than gB (23–25), and current CMV vaccines largely fail to induce epithelial entry-specific neutralizing Abs to levels seen in healthy donors (26). As a consequence, the focus of vaccine development has now shifted to include components of the pentameric complex, such as gH and gL, which as a heterodimer gH/gL in conjunction with gB are essential for viral entry into the cell. These proteins play important roles in viral cell attachment, cell-to-cell spread, and fusion with the cell membrane. Indeed, lack of any one of these components abrogates initiation of the fusion process (27, 28), and studies in a guinea pig model have demonstrated the ability of an Ab directed against gH/gL to protect against congenital CMV infection (29).

Recent progress, however, suggests that protection against CMV-related disease requires both humoral and cellular immunity. Therefore, the ability to induce both has been recognized as an important attribute for an optimal vaccine candidate (30). Screening of the viral proteome identified gB as the most immunodominant CD4 T cell target from 213 CMV open reading frames (31). This study did not investigate individual peptide epitopes although a limited number of epitopes have now been described. Whereas CD8^+^ T cell epitopes have also been identified, most are restricted through HLA class II alleles (32–36). Of these, the HLA DRB1*0701 (DR7)–restricted peptide epitope DYSNTHSTRYV (DYS) is of particular interest, as it induces arguably the largest CD4^+^ T cell response observed against a pathogen to date (37). Up to 16% of the total CD4^+^ T cell pool can be directed against this single viral epitope, and the TCR usage for this particular epitope is highly conserved between individuals (38). Furthermore, following endogenous synthesis gB epitopes are presented efficiently at the cell surface within HLA class II. This efficient access into the MHC class II pathway stems from a sorting sequence in the C-terminal region of gB that directs it into the endosomal compartment of the cell, therefore providing direct access to the HLA class II Ag-processing pathway (39). This targeting may explain the immunodominance of this glycoprotein, as it allows direct presentation of the Ag for CD4^+^ T cell recognition by infected cells without the need for cross-presentation by professional APCs. Despite these findings, detailed studies about individual gB-derived epitopes are missing. Moreover, very little is known about the cellular immune response against the other two major CMV glycoproteins, gH and gL (37, 40) although it has been suggested that these proteins may also represent good CD4^+^ T cell targets as, such as gB, they localize to the *trans*-Golgi network (TGN)/endosomal compartment, which acts as the site of virus assembly in infected cells. As such, gH and gL protein could have relatively easy access to the HLA class II pathway.

In this study, we have undertaken a comprehensive analysis of the T cell immune response to three major CMV-derived glycoproteins, gB, gH, and gL. The aim was to determine the relative immunodominance of the glycoproteins, identify novel epitopes within each protein, and investigate potential mechanisms that might underlie this pattern. This work is very important in relation to design of the appropriate components within a future CMV vaccine formulation. Our results reveal a remarkable difference in the cellular immunodominance of gB in comparison with gH and gL, which correlates with the pattern of cellular localization and ability to undergo direct endogenous presentation to CD4^+^ T cells. Moreover, the data suggest that subunit components of gH and gL may represent poor components for a CMV vaccine unless approaches such as protein conjugation are undertaken to provide T cell help to support generation of high-affinity humoral immunity.

## Materials and Methods

### Subjects

Seventeen healthy CMV-seropositive donors and one CMV-seronegative donor were recruited into the study. Supplemental Table I contains information about donor age and HLA class II genotype. Ethical permission was granted by the West Midlands (Black Country) Research Ethics Committee (REC 07/Q2702/24), and all donors gave written informed consent.

### Peptide libraries of CMV-derived glycoproteins

To screen for T cell responses specific for three CMV glycoproteins gB, gH, and gL, peptide libraries were purchased from Alta Biosciences. Each library consisted of sequential peptides 20 aa in length, overlapping by 15 aa and spanning the whole sequence of the protein. For gB (GenBank/Swiss-Prot ID CAA35414) the library comprised 179 peptides, for gH (GenBank ID CAA35390.1) 146 peptides were synthesized, and the gL (UniProtKB/Swiss-Prot ID P16832.2) library contained 53 peptides. All individual peptides were dissolved in DMSO and peptide pools were generated for each glycoprotein based on a screening cross-matrix designed as previously described (41). One example for this approach can be found in Supplemental Fig. 1. By doing this, each peptide was contained exactly within two pools, which allowed rapid identification of immunogenic peptide epitopes.

### Isolation and stimulation of PBMCs

Heparinized blood (60 ml) was collected and lymphocytes were isolated by density gradient centrifugation using Lympholyte cell separation media (Cedarlane). To identify virus-specific T cells within the PBMC fraction, 1.5 × 10^6^ freshly isolated cells were resupended in 500 μl RPMI 1640 supplemented with 10% FCS and 1% penicillin/streptomycin. Peptides were added at a final concentration of 5 μg/ml and PBMCs were then incubated overnight at 37°C and 5% CO_2_ in the presence of brefeldin A (10 μg/ml final concentration; Sigma-Aldrich) to block cytokine secretion. Cells stimulated with an equivalent volume of DMSO served as a negative control; PBMCs stimulated with staphylococcal enterotoxin B (final concentration, 0.2 μg/ml; Sigma-Aldrich) functioned as a positive control.

### Intracellular cytokine staining

**FIGURE 1.**
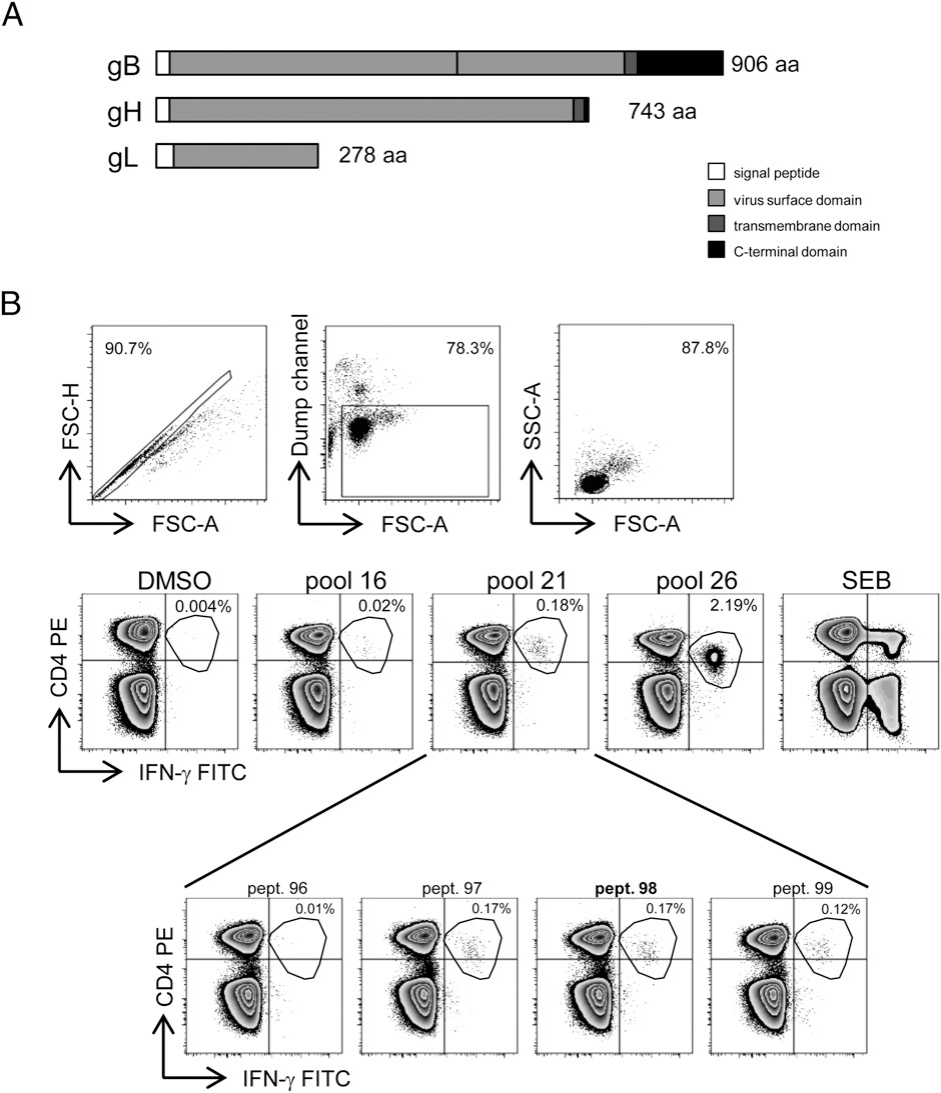
Identification of peptide-specific T cells by intracellular cytokine staining for IFN-γ. (**A**) Schematic representation of the three CMV-derived glycoproteins studied. The identification of a CD4-restricted gB-derived epitope is shown as an example in (**B**). PBMCs were stimulated overnight, then stained with Live/Dead fixable violet stain and anti-CD19 (dump channel) as well as anti-CD4 and anti-CD8. After fixation (4% paraformaldehyde) and permeabilization with 0.5% saponin, activated T cells were identified by staining with anti–IFN-γ. DMSO and staphylococcal enterotoxin B (SEB)–stimulated cells served as negative or positive controls, respectively. Shown are the gating strategy (*top row*) and results of the first round of screening (*middle row*). The individual peptide epitope was then identified in a subsequent screening (*bottom row*). Numbers indicate the frequency of IFN-γ–producing cells within the total CD4^+^ T cell population. FSC-A, forward scatter area; FSC-H, forward scatter height; SSC-A, side scatter area.

Following overnight stimulation, activated T cells were identified by flow cytometric detection of intracellular IFN-γ expression. For this, cells were washed with 1× PBS and stained with Live/Dead fixable violet stain (Invitrogen) for 15 min at room temperature (RT) followed by one wash in 1× PBS and one in staining buffer (1× PBS plus 0.5% BSA and 2 mM EDTA). T cells were then identified by staining with anti–CD4-PE (BD Biosciences) and anti–CD8-PECy5 (Beckman Coulter), and B cells were excluded by staining with anti-CD19 Pacific Blue (eBioscience; dump channel). Cells were incubated for 15 min at 4°C before washing off excess Ab with staining buffer. Fixing was carried out with 4% paraformaldehyde (in PBS; Sigma-Aldrich) for 15 min at RT before permeabilizing with 0.5% saponin (in PBS; Sigma-Aldrich) for 5 min. Intracellular IFN-γ was then stained with an anti–IFN-γ FITC Ab (BD Biosciences) followed by a final wash in staining buffer. Acquisition was carried out on an LSR II flow cytometer and FACSDiva software (BD Biosciences) collecting 300,000 live lymphocytes, and data were analyzed using FlowJo software version 7.6.5 (Tree Star). For the analysis, doublets were excluded based on forward scatter height versus forward scatter area, followed by exclusion of dead cells and CD19^+^ cells. The lymphocyte population was then gated on a forward scatter area versus side scatter area scatter plot (see Fig. 1B), and the proportion of IFN-γ–producing cells was determined within the CD4 or CD8 T cell subset. A cytokine response was defined as positive when the frequency of cytokine-producing cells was at least 2-fold increased above background frequency detected in the corresponding DMSO-stimulated sample.

For the extended functional analysis of glycoprotein-specific T cells, PBMCs were stimulated as described above, stained with Live/Dead fixable violet stain (Invitrogen), followed by surface staining with anti-CD19 (to exclude B cells; eBioscience), anti–CD3-AmCyan, and anti–CD4-PE-CF594 (both BD Biosciences). Following fixation and permeabilization, cells were then stained for intracellular IFN-γ FITC (BD Biosciences), TNF-α PE-Cy7 (eBioscience), IL-2 PE (BioLegend), and a marker for cytotoxic potential granzyme B (GzmB) Alexa Fluor 647 (BioLegend). Acquisition was carried out as above. Analysis was done using FlowJo version 7.6.5 and SPICE version 5.3 (42). For analysis, single, live, CD19^−^ lymphocytes were gated. Of these, CD3^+^ cells were identified and CD4^+^ T cells were gated on. Using Boolean gating, all combinations of responding cells were determined using IFN-γ, TNF-α, IL-2, and GzmB as read-out parameters and background (DMSO control) was substracted.

### T cell cloning

Once peptide epitopes were identified, a selection of CMV-specific CD4^+^ T cells were cloned to identify HLA restriction and to be used in further experiments. PBMCs were isolated as above and CD8^+^ T cells depleted using magnetic bead selection (Dynal) according to the manufacturer’s instructions. Epitope-specific T cells were enriched with an IFN-γ secretion assay cell enrichment and detection kit (Miltenyi Biotec) following a 3-h peptide stimulation. This was done according to the manufacturer’s recommendations. Selected IFN-γ–producing cells were then cloned by limiting dilution in RPMI 1640 plus 5% human serum plus 1% penicillin/streptomycin and seeded with PHA-treated, gamma-irradiated buffy cells as well as peptide-loaded, gamma-irradiated autologous lymphoblastoid cell lines (LCLs). After 3 d, cultures were fed with RPMI 1640 containing 60% MLA144 supernatant, 5% human serum, 1% penicillin/streptomycin, and 100 U IL-2. Ag specificity of expanding cells was confirmed at ∼14 d. For this either 5 × 10^4^ peptide-loaded or DMSO-pulsed autologous LCLs were cocultured overnight with expanded T cells, and IFN-γ was detected in the supernatant by ELISA as described below. For further expansion, cells were cultured in RPMI 1640 containing 30% MLA144 supernatant, 10% FCS, 1% human serum, 1% penicillin/streptomycin, and 50 U IL-2 and occasionally restimulated with peptide-loaded, gamma-irradiated LCLs, and gamma-irradiated feeder cells were added at the same time.

### IFN-γ ELISA

To detect activation of virus-specific T cells, these were cocultured with Ag-loaded cells (fibroblasts or LCLs) and supernatants were analyzed for secreted IFN-γ using an ELISA (Thermo Scientific). Briefly, plates were coated overnight with anti–IFN-γ Ab followed by a blocking step with 1% BSA in 1× PBS and 0.05% Tween 20. Culture supernatants were then added to the plate and incubated for 2 h at RT followed by a wash step with PBS–Tween 20. Bound IFN-γ was detected with a secondary biotinylated Ab for 1 h at RT, followed by a wash step and addition of ExtrAvidin-peroxidase (Sigma-Aldrich). Substrate solution was added, the colorimetric reaction stopped with 1 M HCl, and absorbance was measured at 450 nm.

### HLA restriction analysis of T cell responses

To identify the HLA restriction of newly identified T cell epitopes, 2000 clonal T cells were cocultured overnight at 37°C and 5% CO_2_ in V-bottom plates with 5 × 10^4^ peptide-loaded LCLs, which were either autologous or partially HLA matched. DMSO-pulsed LCLs and T cells alone served as controls. In some cases peptide-loaded LCLs were preincubated with anti–HLA-DR or anti–HLA-DQ Abs for 2 h at 37°C and 5% CO_2_ before addition of the T cells. T cell activation was determined by detection of IFN-γ in the culture supernatant using ELISA as described above.

### T cell recognition of virus-infected cells

Fibroblasts expressing the restricting HLA allele were seeded at 1.5 × 10^4^ cells per well in a 96-well plate and treated with IFN-γ for 72 h to induce HLA class II expression. Cells were then infected at a multiplicity of infection of 1 with a purified preparation of live CMV or UV-inactivated virus (Merlin wild-type [WT] strain) and left for 24 h, then washed with PBS before addition of 20,000 T cells. Peptide-loaded fibroblasts served as a positive control, and uninfected fibroblasts and T cells alone served as negative control. After overnight incubation at 37°C and 5% CO_2_ supernatants were analyzed for IFN-γ production by ELISA (see above).

HLA-matched fibroblasts were not available for both epitopes, and autologous PBMCs therefore served as target cells. PBMCs (only monocytes are permissive for the virus) were infected with CMV at a multiplicity of infection of 1 for 24 h, then washed with PBS and 1 × 10^5^ infected cells were cocultured with 5000 T cells in a 96-well V-bottom plate. Uninfected PBMCs served as a negative control. IFN-γ was detected in the supernatant following 18 h of coculture at 37°C and 5% CO_2_ using ELISA.

### Plasmids and transfection

To express the gB-V5 and gH-V5 proteins, the human CMV (HCMV) gB (UL55) and gH (UL75) genes were subcloned into the pcDNA3.1-V5 plasmid vector. For the expression of GFP fusion protein, the HCMV gB and gH genes were then sublconed into pd2EGFP-N1 vector, in which the GFP is fused to the C terminus of the glycoprotein. All constructs were verified by restriction digest and sequence analysis. Transient transfection of human embryonic kidney (HEK)293 cells (American Type Culture Collection) and MelJuSo (MJS) cells (a human melanoma–derived cell line) (43) with plasmid DNA was routinely performed using Lipofectamine 2000 (Invitrogen) according to the manufacturer’s instructions.

### Western blot analysis of protein expression

To confirm that proteins were expressed, HEK293 cells were transiently transfected with the glycoprotein constructs using Lipofectamin 2000 according to the manufacturer’s recommendations. Twenty-four hours later cells were harvested and lysates generated by sonication. Proteins of a 20-μl sample were separated by SDS-PAGE using Novex 4–12% Bis-Tris gels and MOPS running buffer (Invitrogen) and then transferred onto a polyvinylidene difluoride membrane (Invitrogen) by electroblotting. Following a blocking step (5% milk in PBS–Tween 20) CMV glycoproteins were detected by an anti-V5 Ab (primary Ab; Invitrogen) and an anti-mouse peroxidase-conjugated secondary Ab (Sigma-Aldrich). Detection was done using an ECL detection kit (GE Healthcare) following the manufacturer’s instructions for developing and a ChemiDoc MP imaging system (Bio-Rad). ImageLab software was used to quantify expression levels of the proteins analyzed.

### T cell recognition of Ag taken up exogenously

After quantification as described above, equal amounts of each glycoprotein were fed to autologous LCL overnight in Opti-MEM media at 37°C and 5% CO_2_. Excess lysate was washed off and 1 × 10^5^ LCLs were cocultured with 5000 clonal T cells overnight in RPMI 1640 plus 10% FCS and penicillin/streptomycin before IFN-γ content was determined in the culture supernatant by ELISA (as above).

### T cell recognition of endogenously processed Ag

Autologous LCLs were transfected by electroporation (settings at 270 V and 950 μF) in Opti-MEM media, then transferred into RPMI 1640 plus 10% FCS and penicillin/streptomycin for 24 h. Dead cells were removed by density gradient centrifugation with Lympholyte cell separation media (Cedarlane) before use in the assay. To analyze T cell recognition, 5000 clonal T cells were cocultured overnight in V-bottom plates with 1 × 10^5^ transfected LCLs at 37°C and 5% CO_2_. Supernatants were harvested and analyzed for IFN-γ by ELISA as described above.

### Confocal microscopy

MJS cells were seeded onto coverslips and transiently transfected with GFP-expressing constructs the following day using Lipofectamine 2000 according to the manufacturer’s recommendations. Twenty-four hours later cells were fixed and permeabilized with methanol/acetone (1:1) for 20 min at −20°C. Following a blocking step, costaining with a mouse anti–HLA-DMα Ab (Abcam) for 2 h at RT was carried out. Subsequently, slides were washed with 1× PBS before addition of an anti-mouse IgG1 Alexa Fluor 594 Ab for 1 h at RT. Following another wash step with 1× PBS, coverslips were mounted onto microscope slides with DAPI containing Vectashield mounting media (Vector Laboratories) and sealed. Analysis was done on a laser scanning microscope (LSM) 510 confocal microscope and LSM image browser (Zeiss).

## Results

### gB induces strong CD4^+^ T cell responses against a wide range of peptide epitopes

gB is an immunodominant CD4^+^ T cell target (31) but only a limited number of HLA class II–restricted T cell epitopes have been identified from within the protein, and cellular immunity against other glycoproteins has not been investigated in detail. We chose to investigate T cell immune responses against gH and gL, as these glycoproteins are abundant within the viral envelope and form a major component of the pentameric complex, which is currently a focus of vaccine development.

gB is a type I transmembrane protein, 906 aa in length, and contains a furin cleavage site at position 459 and a long cytoplasmic domain of 136 aa (Fig. 1A). gH is also a type I transmembrane protein and at 743 aa in length it is 82% of the length of gB with a C-terminal domain of only 7 aa. gL is much smaller at 278 aa, 30% the size of gB, and is anchored in the viral envelope by its close association to gH but itself lacks a transmembrane domain.

Peptide pools of each protein were designed using a cross-matrix such that each peptide was contained in exactly two of the pools (41). This approach allows rapid identification of antigenic peptides. As an example, the gB pools 14–27 contained overlapping peptides consecutively spanning the whole sequence of the protein, whereas pools 1–13 were made up of every 13th peptide in the sequence (Supplemental Fig. 1). In the same fashion, peptide pools were generated for both gH and gL, with the former screening matrix consisting of 25 pools and the latter of 15 pools.

Peptide-specific T cells were identified by flow cytometric analysis of intracellular cytokine production following stimulation of PBMCs with the peptide pools (intracellular cytokine staining [ICS]). IFN-γ was used for analysis, as the vast majority of CMV-specific T cells have been shown to produce this cytokine in response to Ag stimulus (44). ICS allowed simultaneous detection of CD4^+^ and CD8^+^ T cells and was highly sensitive. In particular, T cell responses as low as 0.008% of the T cell subset were readily identifiable using this approach, and experimental criteria are described in *Materials and Methods*. Fig. 1B shows a representative example of CD4^+^ T cell responses detected in one donor, ranging from 0.02 to 2.19% of all CD4^+^ T cells induced by one pool of peptides used to stimulate the PBMCs. In cases where an immune response was detected against an individual peptide pool, PBMCs were subsequently stimulated with individual peptides from the pool to identify specific epitope responses (Fig. 1B).

**FIGURE 2.**
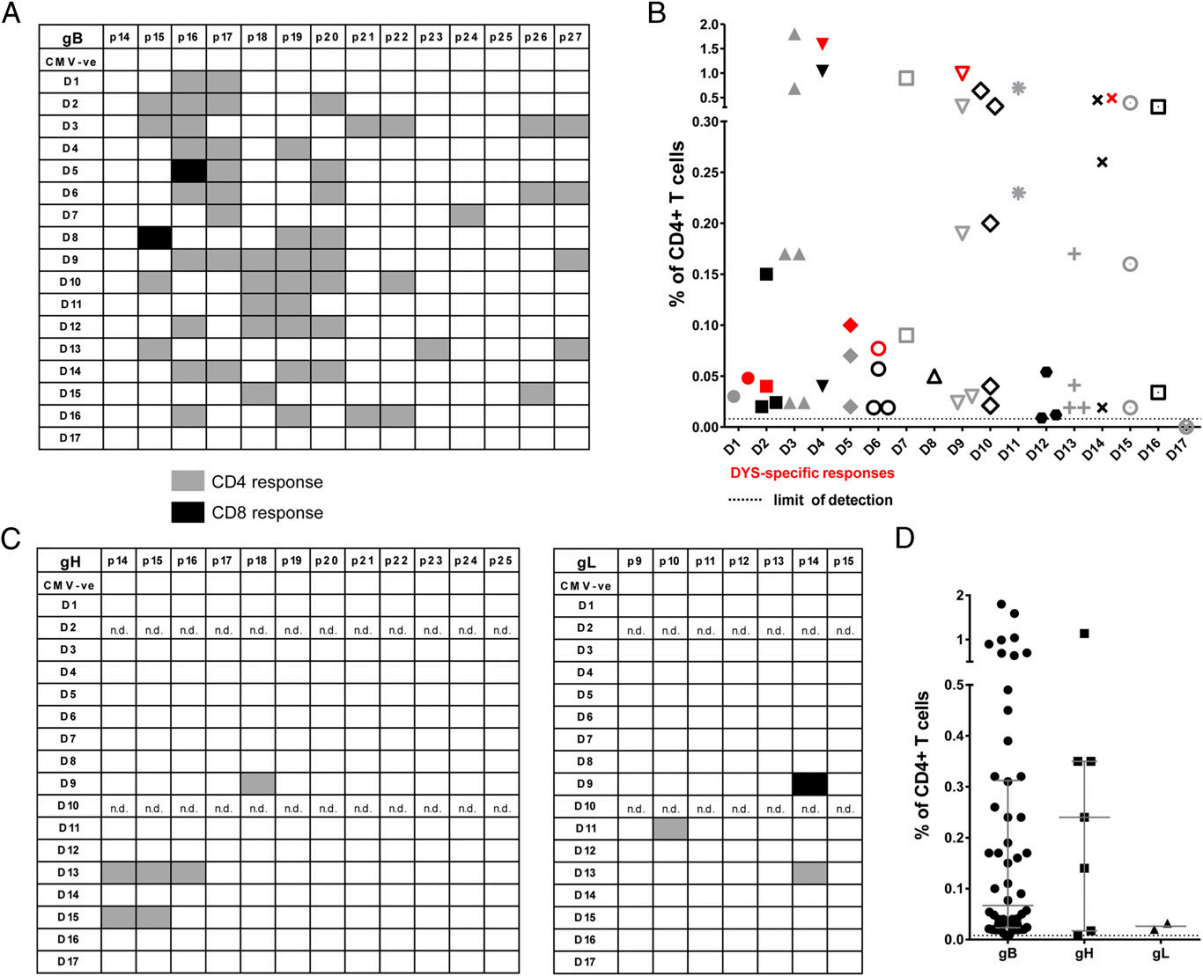
T cell responses are much more frequent against gB than against gH and gL. Summary of gB screening results (**A**). With the exception of one donor, T cells producing IFN-γ in response to stimulation with peptides derived from gB could be detected in all CMV-seropositive subjects tested. Peptide epitopes can be found along most of the protein sequence, consecutively contained in pools 14–27. The T cell response to gB varies greatly in magnitude and number of epitopes recognized (**B**). The proportion of CD4^+^ T cells responding to individual gB-derived epitopes is shown for each donor. Up to six peptide epitopes were identified per donor. DYS-specific responses, which have been previously reported, are highlighted in red. In comparison, T cell responses to gH and gL are much less frequent as summarized in (**C**). The response magnitudes were comparable to those against gB (**D**). Individual T cell responses are shown as a proportion of total CD4^+^ T cells for each of the glycoproteins studied. Each bar represents the median CD4^+^ T cell response size with interquartile range.

PBMCs from 17 CMV-seropositive donors were screened for T cell reactivity against gB (Supplemental Table I). gB-specific CD4^+^ T cells were identified in all but one of the donors (Fig. 2A). No responses were observed in CMV-seronegative donors. Peptide epitopes were derived from along the majority of the protein sequence, but interestingly they were not detectable from the first 80 aa (pool 14) or the transmembrane domain (contained in pool 25). Each donor responded to at least two peptide epitopes, with a maximal response to five peptides in donor 3. The vast majority of these T cell responses were restricted through CD4, but also two CD8-restricted T cell responses were identified (donors 5 and 8). In the subscreening, generally one to three consecutive peptides were able to elicit T cell reactivity. Of those, the single peptide capable of inducing the largest IFN-γ response was defined as the peptide epitope (Fig. 1B), although minimal peptide epitopes have not been further characterized so far. Using this approach, 29 individual peptides were identified to contain CD4^+^ T cell epitopes, of which only 5 have previously been reported (Supplemental Table II). Just two CD8-restricted T cell epitopes were identified, both of which have not been previously known (Supplemental Table II). The magnitude of the gB-specific CD4^+^ T cell response ranged from 0.06 to 2.88% of the total CD4^+^ T cell population, with a maximal response of 1.8% against one single epitope (Fig. 2B). Unsurprisingly, DYS was not the only epitope capable of inducing such a large T cell response. In 9 of 17 donors, >0.3% of all CD4^+^ T cells were specific for one single epitope, and in most cases this was not the only epitope per donor. A median of 1.22% (range, 0.34–2.88%) of all CD4^+^ T cells were directed against gB in those individuals. Apart from one HLA-DR7^+^ donor, the T cell response to the known HLA-DR7–restricted epitope DYS was the largest one observed in these individuals, but it did not always reach the extremes previously observed in elderly donors (38). In the remaining seven donors, <0.15% of CD4^+^ T cells were specific for one single epitope, but most displayed T cells specific for more than one single epitope (between one and four) with a median response size of 0.17% (range, 0.05–0.25%) of all CD4^+^ T cells. This was independent of the donor age.

### gH and gL contain far fewer T cell epitopes than does gB

It has been speculated that, similar to gB, the other CMV glycoproteins would also represent very good targets for CD4^+^ T cells, based on the fact that the TGN is the site of virus assembly in infected cells (45). Therefore, we undertook a detailed analysis of the T cell response to gH and gL, as these are the two other conserved major glycoproteins contained in the viral envelope and the pentameric complex.

Using the same peptide cross-matrix approach, 15 of the 17 donors screened for gB responses were tested for responses against gH and gL using ICS. In contrast to gB, gH-specific CD4^+^ T cells were only detected in three donors and no CD8^+^ T cell responses were observed (Fig. 2C). The magnitude of these responses ranged from 0.008 to 1.14% of the total CD4^+^ T cell population (median, 0.24%). The frequency of T cell responses against gL was comparable in that responses were again observed in only three donors (Fig. 2C). Two of these were CD4-restricted T cell epitopes (0.02–0.03% of CD4^+^ T cells), whereas the third was restricted through CD8 (0.014% of total CD8^+^ population). Interestingly, although far fewer T cell epitopes are contained within the gH and gL proteins, the magnitude of those responses identified was comparable to those against gB. Indeed, when the magnitude of the T cell response against gB is compared with that directed against gH or gL, respectively, no significant difference was observed in the median response size (Fig. 2D). The peptides identified to contain T cell epitopes, and the magnitude of the T cell response, are both summarized in Supplemental Table II.

### gB-specific T cells display a distinct pattern of polyfunctional capacity following antigenic stimulation

In addition to examining the magnitude of the T cell response against gB, gH, and gL, we next examined the functional profile of CD4^+^ T cell responses against the three glycoproteins in more detail. The individual and combinatorial patterns of IFN-γ, TNF-α, IL-2, and GzmB expression was determined to assess the polyfunctional profile of the virus-specific cells. This analysis was performed for T cells recognizing newly identified gB-derived epitopes (*n* = 7) and also for those specific for all epitopes identified within gH (*n* = 7) and gL (*n* = 2).

**FIGURE 3.**
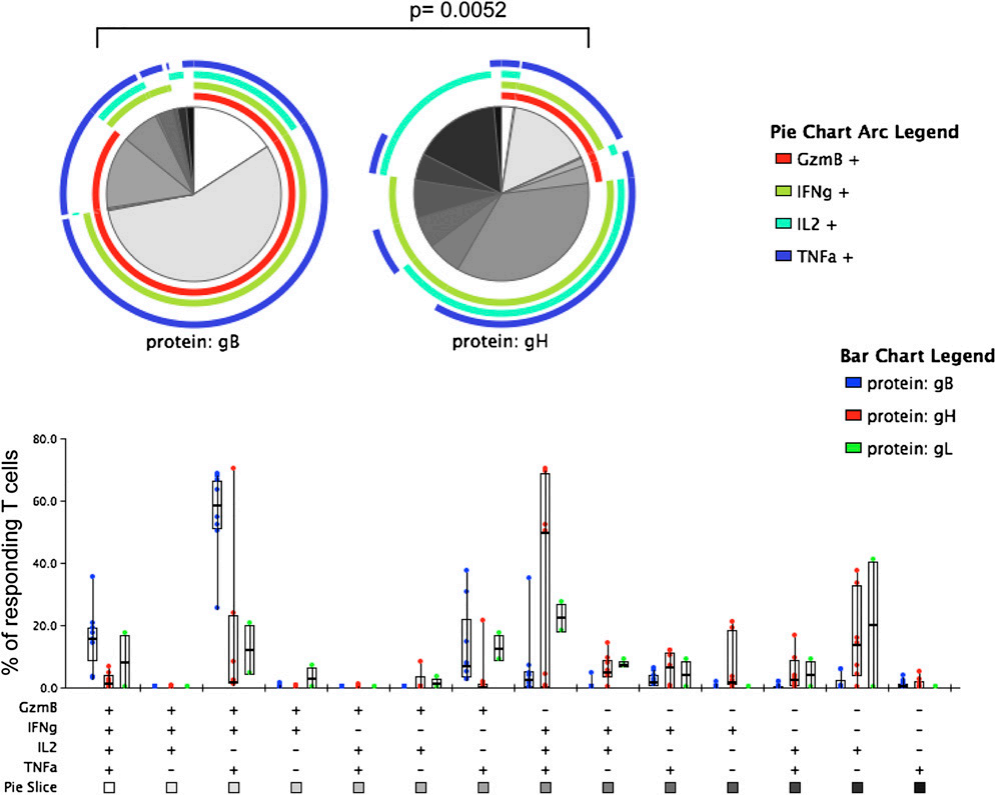
Glycoprotein-specific T cells vary in their functional capacities. Functional characterization of glycoprotein-specific T cells was carried out following peptide stimulation of PBMCs using multicolor flow cytometric analysis, and a Boolean gating strategy was applied. Bar charts represent individual data for responses against each of the three glycoproteins studied. Shown are box-and-whisker plots representing median values with interqartile range and minimum and maximum values. Pie charts summarize the data for gB and gH and represent the proportion of responding cells with cytotoxic potential and/or single, double, or triple cytokine production capacity.

gB-specific CD4^+^ T cells displayed a strongly cytotoxic phenotype (87% GzmB^+^) following peptide stimulation with very high levels of TNF-α and IFN-γ production (Fig. 3). Only 26% of responding cells expressed IL-2, although 16% exhibited a complete polyfunctional profile by expression of all four markers. In contrast, gH-specific T cells exhibited a markedly reduced cytotoxic profile, with GzmB expression observed in only 26% of cells, although it was noteworthy that 59% of gH-specific T cells retain the capacity to produce IL-2. T cell responses against the two gL-derived epitopes were comparable to those seen for gH-specific T cells (Fig. 3, bar charts).

### Glycoprotein-specific CD4^+^ T cells display a broad range of HLA restrictions and have moderate affinity for Ag

To investigate why other glycoproteins besides gB were poor CD4 T cell targets, we prepared CD4^+^ T cell clones specific for representative epitopes in all three proteins. Using IFN-γ capture enrichment and cloning by limiting dilution, T cell clones specific for five epitopes derived from gB and one each from gH and gL were generated. The isolation of CD4^+^ T cell clones allowed determination of the HLA restriction of these new epitopes, assessment of functional avidity of the T cell clones, and investigation of whether and when the epitopes are generated during the course of natural infection. Subsequently, the epitopes are named using the first three amino acids of the respective peptide sequence.

**FIGURE 4.**
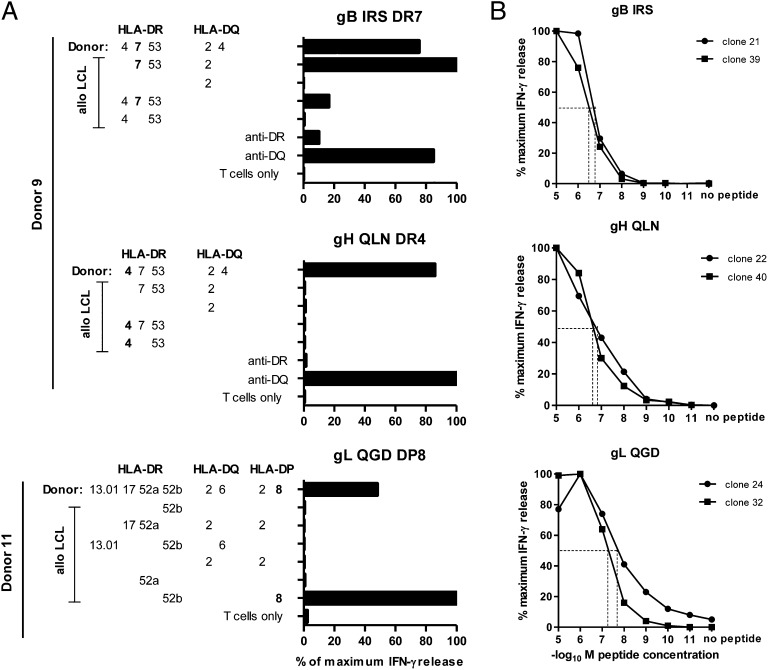
HLA restriction and functional avidity of CD4^+^ T cell clones specific for gB, gH, and gL. Representative examples of HLA restriction assays: clonal T cells were cocultured with either autologous or partially HLA-matched peptide-loaded LCLs, and IFN-γ was detected in the supernatant to analyze T cell activation (**A**). The functional avidity of CD4^+^ T cell clones specific for the same epitopes was investigated by preincubation of autologous LCLs with decreasing concentrations of the peptide, followed by measurement of IFN-γ release after incubation with T cells (**B**). Each experiment was carried out at least twice in duplicates.

Table I lists the protein, peptide sequence, and HLA restriction of all seven cloned T cell responses. The HLA restriction of each clone was determined with the use of LCLs. Clones were tested for recognition of peptide-loaded autologous LCLs and also allogeneic, peptide-loaded LCLs that were partially matched for specific HLA class II alleles. Three examples from this analysis are shown in Fig. 4A. The HLA restriction of both the gB-derived epitope IRS and gH-derived epitope QLN was defined with T cell clones from donor 9, and gL-derived epitope T cell clones were generated from donor 11. Peptide IRS was presented only by LCLs sharing HLA-DR7, thus identifying this as the restricting allele. The QLN response was analyzed in the same donor using the same panel of partially matched LCLs. T cell recognition was blocked completely by preincubation with an anti–HLA-DR Ab, however none of the allogeneic LCLs induced T cell recognition. As the DR7 restriction of IRS-specific clones from the same individual was clearly identified using this panel of LCLs, we concluded that this epitope is HLA-DR4 restricted. Several subtypes of HLA-DR4 have been observed, and polymorphisms occur in regions of the molecule that play a role in the interaction of the TCR and its Ag (46, 47). This may explain why this particular epitope was only presented by autologous LCLs. HLA-DR4 and -DR7 are both extremely closely linked to DR53, but no such subtype-specific differences have been reported for this HLA allele, supporting our conclusion that the QLN epitope is HLA-DR4 restricted. The final example shows the HLA restriction of the gL-derived epitope QGD, which was determined to be HLA-DP8.

**Table I.** Protein origin, peptide sequence, and HLA class II restriction of newly identified CD4^+^ T cell epitopes

Protein	Peptide Sequence	HLA Restriction
gB	**RSY**AYIYTTYLLGSNTEYVA	HLA-DR7
	**NAS**YFGENADKFFIFPNYTI	HLA-DP2
	**LTF**WEASERTIRSEAEDSYH	HLA-DP2
	**IRS**EAEDSYHFSSAKMTATF	HLA-DR7
	**NEQ**AYQMLLALARLDAEQRA	HLA-DR52b
gH	**QLN**RHSYLKDSDFLDAALDF	HLA-DR4
gL	**QGD**KYESWLRPLVNVTRRDG	HLA-DP8

Epitopes are named using the first three amino acids of their peptide sequence, as highlighted in bold.

The functional avidity of T cell clones specific for the three peptide epitopes was determined by peptide titration analysis (Fig. 4B). Autologous LCLs were loaded with decreasing concentrations of the epitope peptide, and functional avidity was defined as that peptide concentration that induced a half-maximal IFN-γ response. This value was very similar for IRS- and QLN-specific T cell clones, with ∼300 nM. The QGD-specific T cell clones displayed a slightly higher avidity of ∼50 nM. These values are comparable to values of other virus-specific CD4^+^ T cells (48, 49).

### Glycoprotein-specific T cell clones recognize CMV-infected target cells and do not require de novo protein synthesis

**FIGURE 5.**
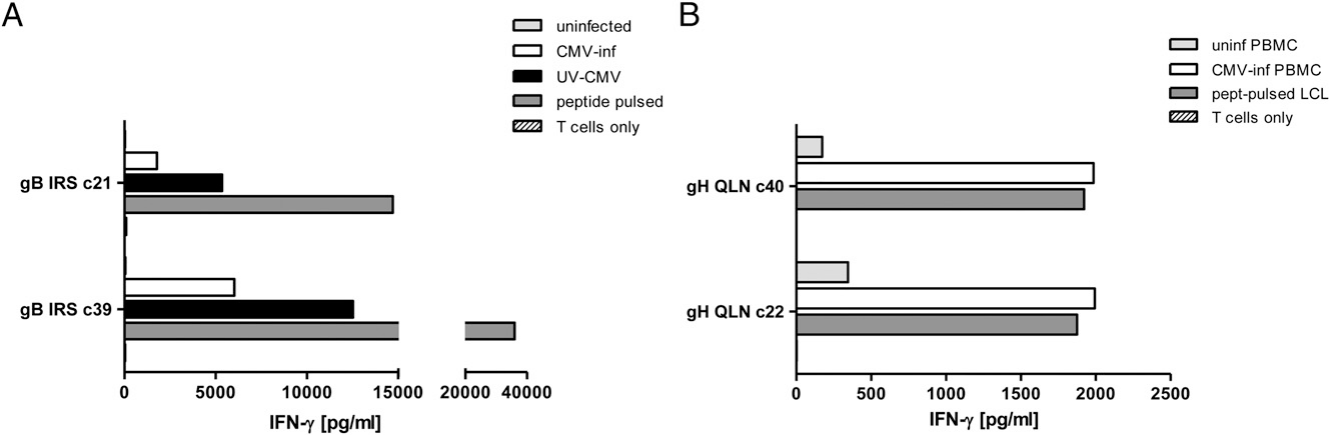
Newly identified T cell epitopes are generated during natural infection without the need for de novo synthesis. To determine whether glycoprotein-derived epitopes are generated in the context of viral infection, HLA-matched fibroblasts, pretreated with IFN-γ (**A**) or autologous PBMCs (**B**), were infected with purified CMV for 24 h prior to addition of clonal T cells specific for peptides IRS or QLN, respectively. T cell activation was detected by analysis of IFN-γ in supernatants using ELISA. Shown is a representative of two experiments for each epitope studied.

Another major incentive for generating T cell clones was to use these to confirm that the individual peptide epitopes were presented during the course of natural viral infection of a target cell. In case of the DR7-restriced epitope IRS, HLA-matched fibroblasts were treated with IFN-γ to induce HLA class II expression and then infected with a purified preparation of CMV (Merlin strain) for 24 h prior to addition of T cell clones. Recognition of the Ag was determined by detection of IFN-γ within the culture supernatant. Peptide-loaded and uninfected fibroblasts served as controls. Results show that T cells were indeed able to recognize virus-infected fibroblasts (Fig. 5A). Furthermore, infection of fibroblasts with a UV-inactivated CMV preparation established that de novo protein synthesis was not necessary, showing that incoming virus particles contained sufficient amounts of gB to sensitize specific T cells. For the gH-derived epitope QLN, no HLA-matched fibroblasts were available and we therefore infected autologous PBMCs with virus, as monocytes are permissive for infection. This confirmed that the peptide epitope QLN was also generated efficiently in the context of a viral infection (Fig. 5B).

### Peptide epitopes from gH are not presented following endogenous protein synthesis, and this is not overcome by addition of the C-terminal domain from gB

**FIGURE 6.**
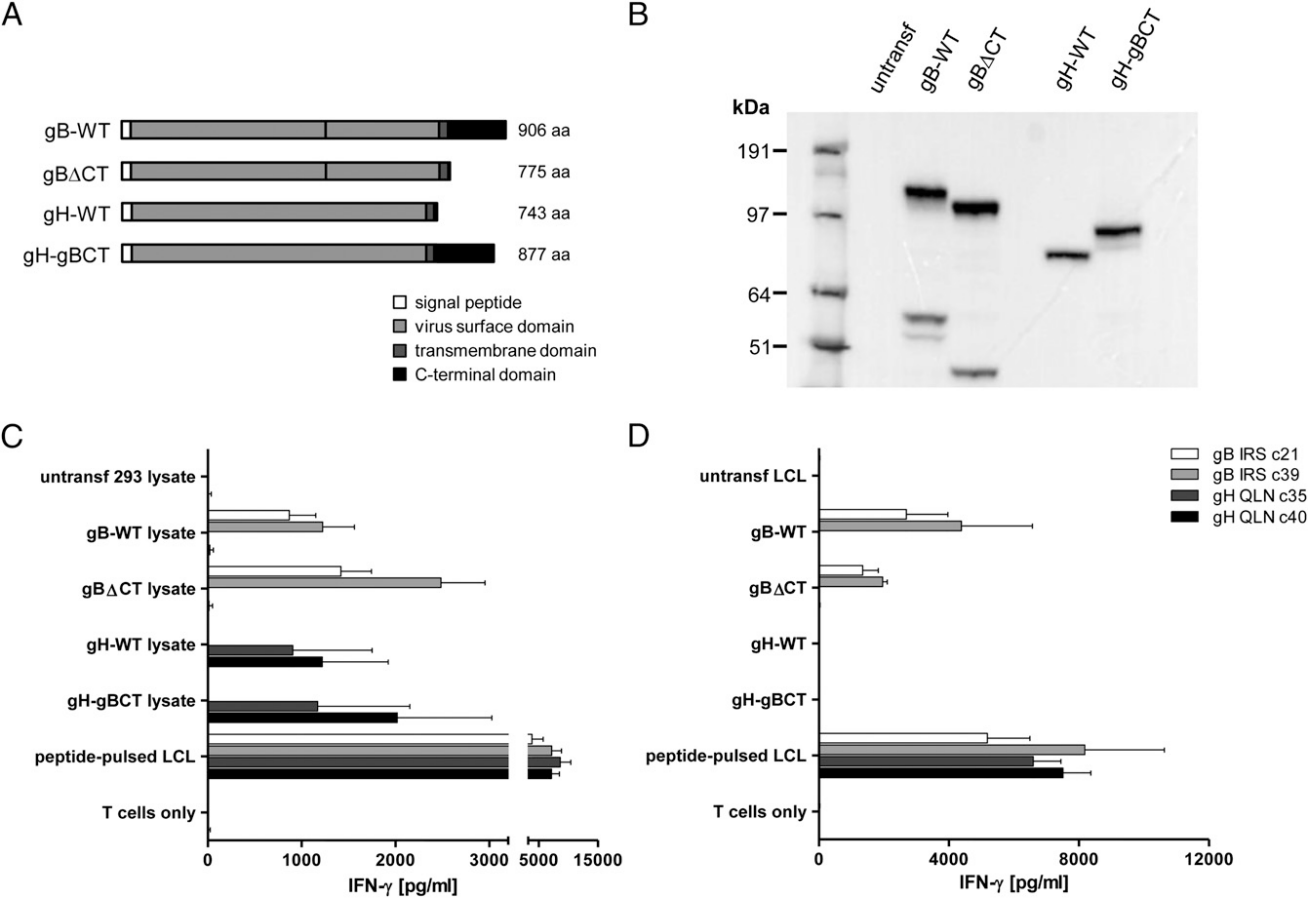
gB and gH induce different patterns of T cell recognition. gB-WT protein was truncated after the transmembrane domain (gBΔCT), and correspondingly the C-terminal domain of gB was added to the gH-WT protein (gH-gBCT) to analyze its effect on Ag processing and presentation (**A**). Protein expression in HEK293 cells was confirmed by Western blot 24 h following transfection (**B**). T cell recognition of exogenously (**C**) or endogenously (**D**) processed Ag was determined. All four proteins were processed when taken up exogenously by autologous LCLs and presented to CD4^+^ T cell clones, whereas only gB-WT and gBΔCT were processed endogenously for presentation in HLA class II when LCLs were transfected with the target Ag. Graphs in (C) and (D) summarize three independent experiments for both endogenous and exogenous presentation. Shown are means with SEM.

Because the first part of the present study had revealed profound differences between the immunogenicity of gB in relation to gH and gL, we then went on to study Ag processing of CMV glycoproteins. gB and gH were chosen, as they are both transmembrane proteins and similar in size. T cell clones specific for a gB-derived epitope (IRS) and a gH-derived epitope (QLN) were generated from the same donor, and these were selected to investigate suspected differences in Ag processing of the two glycoproteins. Interestingly, peptide dilution analyses showed that the avidity of these clones was very similar. The C-terminal domain of gB has been suggested to contain a sorting sequence directing it into the HLA class II processing pathway, leading directly to endogenous processing for Ag presentation, and therefore bypassing cross-presentation (39). This report also suggested that other CMV glycoproteins might represent good CD4^+^ T cell targets, as the TGN/endosomal compartment of virus-infected cells acts as the site of virus assembly. To address this question, we generated four recombinant glycoprotein constructs to analyze the importance of the potential sorting sequence contained within the C-terminal domain of gB (Fig. 6A). Two of these expressed WT gB (gB-WT) or gH (gH-WT), whereas the third contained a deletion of the C-terminal domain of gB (gBΔCT) and the final vector adjoined this C terminus to WT gH (gH-gBCT). The constructs contained either a C-terminal V5 tag or GFP to allow detection of protein expression.

Initial experiments analyzed classical HLA class II Ag presentation through uptake of exogenous Ag by APCs via endocytosis, followed by processing and presentation at the cell surface in the context of HLA class II. HEK293 cells were transfected with each of the four constructs, harvested after 24 h, and then sonicated to generate cell lysate. Expression of the protein was confirmed by Western blot analysis (Fig. 6B) and quantified by densitometry. Equal amounts of protein were fed to autologous LCLs overnight to allow uptake and processing of the Ag for presentation at the cell surface. T cell recognition was then analyzed by detection of IFN-γ in the supernatant following coculture of target cells with epitope-specific T cell clones. For each epitope, two T cell clones were tested in the assay. LCLs pulsed with lysate of untransfected HEK293 cells and peptide-loaded LCLs served as controls. All four proteins were found to be processed via the exogenous route for Ag presentation with a clear IFN-γ response to LCLs incubated with HEK293 lysate expressing the relevant glycoprotein (Fig. 6C), thus confirming that all four proteins can be processed and presented in the classical way.

We then went on to investigate whether the epitopes could also be generated from endogenous protein production and enter the HLA class II Ag presentation pathway in a nonclassical manner. Autologous LCLs were transfected with the individual constructs using electroporation, and dead cells were removed after 24 h by gradient centrifugation. Protein expression was confirmed by flow cytometric detection of GFP^+^ cells, and cells were then cocultured with T cell clones prior to detection of IFN-γ release using ELISA. As expected, the gB-derived epitope IRS was indeed generated through an endogenous route of presentation with T cell recognition observed from both T cell clones (Fig. 6D). In contrast, the QLN epitope from gH was not presented at the cell surface following endogenous synthesis of the glycoprotein. In the next step we examined the influence of deletion of the C-terminal domain of gB on the efficiency of peptide presentation following electroporation. Surprisingly, deletion of the domain had very little effect on T cell recognition, with only a slight reduction in IFN-γ release following target cell transfection with the gBΔCT construct. Moreover, the addition of the gB C-terminal domain to WT gH did not lead to processing and presentation of the QLN epitope via an endogenous pathway.

### gB and gH exhibit differential cellular localization

The studies above indicate that gB and gH undergo different patterns of Ag processing during viral infection. An important aspect of this will be their localization within the cell, but to date this has only been investigated in detail for gB. We therefore analyzed the cellular localization of both gB and gH, and also studied whether exchange of the gB C-terminal domain between the two proteins would modulate the observed pattern. GFP-expressing constructs were used to visualize the cellular localization of proteins expressed from the gB, gH, gBΔCT, and gH-gBCT vectors.

MJS cells were transfected, fixed, and permeabilized after 24 h using methanol/acetone (1:1). These were then counterstained with DAPI and mounted on microscope slides for analysis on an LSM 510 confocal microscope. Glycoprotein B expression was contained exclusively to vesicular structures throughout the cytoplasm of the cells (Supplemental Fig. 2, *top left*), in agreement with previous studies. In contrast, gH was distributed evenly within the cytoplasm and did not show any vesicular localization (Supplemental Fig. 2, *bottom left*). Removal of the C-terminal domain from gB (gBΔCT) broadened expression of the protein to include both vesicular and cytoplasmic distribution of the protein. However, when this domain was added to gH, in the gH-gBCT construct, the expression of gH remained solely within the cytoplasm of the cell, in a distribution very similar to that seen for WT gH (Supplemental Fig. 2). These findings support our preceding experiments investigating T cell recognition of endogenously processed Ag, as addition of the gB C-terminal domain to the gH protein does not alter its cellular localization or pattern of Ag presentation.

**FIGURE 7.**
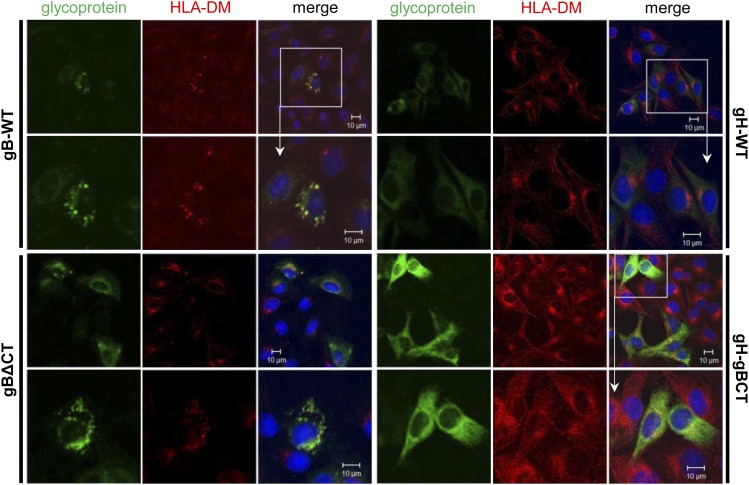
gB protein colocalizes with the HLA class II loading compartment, but gH does not. MJS cells were transfected with GFP-expressing glycoprotein constructs, fixed, and costained with anti–HLA-DM (red). They were then counterstained with DAPI and mounted on microscope slides for analysis. gB-WT protein was contained exclusively within vesicular structures colocalizing with HLA-DMα (*top left*). In cells transfected with gH-WT, the glycoprotein was distributed evenly throughout the cytoplasm, whereas HLA-DM showed punctate staining concentrated particularly in perinuclear regions (*top right*). The same pattern was observed for gH-gBCT (*bottom right*). gBΔCT protein expression was observed broadly within the cell cytoplasm but also retained vesicular localization with relative coexpression with HLA-DM staining (*bottom left*). Scale bars, 10 μm.

HLA-DM is expressed in the HLA class II loading compartment, and we next went on to costain the pattern of glycoprotein expression with an Ab against HLA-DMα. This revealed that the vesicular localization of gB-WT coincided directly with HLA-DM expression and confirms that gB was indeed present within the HLA class II loading compartment (Fig. 7, *top left*). Expression of the gBΔCT protein was also retained with HLA-DM in vesicular structures, although this was less discrete than with the full gB protein (Fig. 7, *bottom left*). gH-WT protein and gH-gBCT were distributed throughout the cell cytoplasm, whereas intense punctate staining of HLA-DM was seen in the perinuclear regions (Fig. 7, *right*). No difference was observed between the WT and gH-gBCT proteins.

## Discussion

In this study, we have defined the relative immunodominance of three of the glycoproteins embedded in the envelope of CMV in eliciting a T cell response and have related this to their mechanism of Ag presentation. gB has been the main focus for a CMV vaccine until recently, but the pentameric complex including gH and gL is now known to be essential for viral entry into endothelial and epithelial cells (22). Moreover, most neutralizing Abs are directed against this complex, which makes it an attractive additional target for vaccine development. A vaccine that combines gB with gH and gL might therefore be of great value in relation to inducing both a neutralizing Ab response and strong T cell immunity. However, detailed study of the T cell response against the gH and gL proteins has not been previously performed, even though structural viral proteins are thought to present good CD4 T cell targets. In both murine CMV (MCMV) and rhesus CMV (RhCMV) models, an important role for CD4^+^ T cells in the control of virus has been implicated (50, 51), and an inverse correlation was observed between the degree of virus shedding, the polyfunctionality index, and proliferative capacities of adult RhCMV-specific CD4^+^ T cells (52). The development of an early proliferative CD4 T cell response in pregnant women undergoing primary CMV infection has also been shown to influence the viral transmission rate (12).

Our initial results revealed major differences between the immunogenicity of gB in contrast to gH or gL. In particular, a T cell response against gB was detectable in 95% of all donors, in contrast to only 20% of individuals in whom T cells specific for gH or gL could be isolated. This observation is not due simply to the relative size of the individual proteins. Indeed, at 743 aa, gH is ∼82% the size of gB, and it is highly abundant within the viral envelope. gL is certainly smaller than the other two proteins, at 278 aa in length, but it is equally abundant (53). For gB, the vast majority of these epitopes were restricted through CD4, but also a small number of CD8-restricted epitopes were identified, in line with previous findings. Every gH-specific T cell response identified in the present study was mediated by CD4^+^ T cells; however, two CD8-restricted epitopes have been published previously (35). A T cell response against gL was rare in our subjects, but it was spread between CD4- and CD8-restricted epitopes, and as only three epitopes in the three donors were identified, firm conclusions about the relative CD4 or CD8 dominance for this protein cannot be made. This is the first time, to our knowledge, that the T cell response against gL has been addressed in detail, and the findings extend observations revealing gB as the most frequently recognized CMV-derived protein, followed by gH and gL (31).

gB is among the most immunodominant of all proteins that have ever been studied. In 41% of individuals the gB-specific T cell response comprised >1% of the total CD4^+^ T cell pool, and this gB-specific repertoire contains a remarkable breadth of peptide responses that had not been previously observed. Indeed, we identified 29 peptide epitopes within the protein, of which only 5 have been reported previously (32, 36, 37). In addition to the breadth of T cell responses, the size of the identified epitope responses can also be substantial. Apart from the DYS peptide previously shown to stimulate very strong CD4^+^ T cell responses, several other gB-derived epitopes were able to induce strong CD4^+^ T cell responses, each representing >0.5% of the total CD4^+^ T cell population. Interestingly, although T cell epitopes were found much less commonly in gH and gL, the magnitude of these individual responses can also be very substantial. Up to 1.14% of all CD4 T cells were found to be specific for the QLN peptide from gH, which indicates that the magnitude of CMV-specific T cell immune responses is not related to the density of peptide epitopes within the target protein. The mechanism that underlies this remarkable immunodominance of CMV proteins is not well understood, but it may relate to recurrent episodes of subclinical viral reactivation or initial priming events.

A key finding was also the marked dominance of CD4^+^ T cell responses against the two larger glycoproteins in comparison with much weaker induction of CD8^+^ T cell responses. CMV-specific CD4^+^ T cells have been less well studied, and only a small number of peptide epitopes have been identified to date. Even so, they play an important role in control of infection through the direct suppression of viral replication and support of virus-specific CD8^+^ T cell function and survival (9, 10, 54). In the murine model of CMV infection, lack of virus-specific CD4^+^ T cells leads to prolonged shedding of the virus from the salivary gland, which is thought to be one of the main routes of horizontal transmission (55). A large proportion of virus-specific T cells are indeed located within the CD4^+^ T cell compartment (31), and virus-specific CD4^+^ T cells can comprise up to 10% of the peripheral CD4 T cell compartment (56). CD4^+^ T cell responses against individual pathogens are often broader and less focused on particular epitopes in comparison with CD8^+^ responses. Nonetheless, hierarchies of immmunodominance can also be discerned in the CD4^+^ T cell compartment. In the context of viral infection, this has been analyzed most in-depth for vaccinia virus and EBV (57, 58). During CMV infection, CD4^+^ T cell immunity seems to be focused on structural proteins, such as tegument components and glycoproteins, which are often expressed late in the virus life cycle, but abundant in incoming virus particles (31, 59). However, there are many factors influencing CD4^+^ T cell immunodominance in addition to Ag abundance and expression kinetics, including HLA restriction element, precursor frequencies of naive T cells, Ag structure, and competition of Ag at the level of APCs (60–63). The stability of the HLA class II/peptide complex seems to play a very important role in determining dominance of individual epitopes (64). Owing to the relatively small cohort size in our study, it is difficult to relate immunodominance of specific epitopes within individuals according to their HLA genotype. However it was notable that all seven donors expressing the HLA-DR*0701 allele displayed a conserved response pattern against gB. Further studies in larger cohorts will be needed to allow firm conclusions about immunodominance hierarchies.

Interestingly, we also observed differences in the functional capacity of T cells in relationship to recognition of the individual glycoproteins. Most notably, T cells recognizing gB-derived epitopes displayed a much greater cytotoxic potential with very strong production of IFN-γ, TNF-α, and GzmB. In contrast, expression of GzmB was substantially reduced in gH- and gL-specific T cells, although it was noteworthy that these cells retained the capacity to produce IL-2, indicating that they were more likely to exert helper function. In terms of polyfunctionality, gB-specific T cells closely resemble the profile previously observed for T cells specific for pp65 and UL86, which are the other two most frequently recognized CD4 T cell targets (44, 65). These observations are also in line with studies from both MCMV and RhCMV model systems where immunodominant proteins drive a predominant Th1-type CD4 T cell response, dominated by production of IFN-γ and TNF-α (59, 66). Studies within HIV infection indicate that polyfunctionality of virus-specific T cells acts as a surrogate marker for protective immunity (67).

With the purpose of addressing the potential mechanisms that may underlie the immunodominance of gB, we generated T cell clones specific for a gB- and a gH-derived epitope from the same donor. All clones used displayed very similar functional avidity. In the first instance we could show that both epitopes were generated in the context of a natural infection in vitro. Rapidly following infection, within 24 h, T cell activation was detected, indicating that the incoming virus particles contain sufficient levels of the glycoprotein to be processed and able to sensitize specific T cells. This is further supported by the fact that the gB-derived epitope could be generated from UV-inactivated virus. Both gB and gH are expressed late in the virus life cycle, but they are abundant in the envelope of virus particles (53, 68). Evidence suggests that gB is directed into the endosomal HLA class II loading compartment, permitting endogenous presentation within virally infected cells (39). This may provide gB with a crucial competitive advantage in terms of access to the Ag presentation pathway compared with other glycoproteins. Having established T cell clones and the fact that both epitopes are generated in the context of a viral infection, we went on to study the mechanisms in more detail. Given that HCMV assembles in endosomal compartments, it had been considered probable that proteins such as gH and gL would also gain access to this mechanism of presentation in virus-infected cells. To address this question, we used the T cell clones specific for the gB- and gH-derived epitopes to study presentation of these epitopes following endogenous or exogenous Ag presentation.

Remarkably, we did not find any evidence for presentation of endogenous gH, whereas T cells did indeed recognize the gB-derived epitope when the proteins were expressed endogenously, confirming its direct access into the HLA class II processing pathway. Importantly, the gH- and gB-derived epitopes were both recognized when Ag was processed following exogenous presentation. To investigate the basis for this difference, we performed confocal analysis to determine the cellular localization of protein expression using constructs containing a GFP tag. As previously reported, gB expression was confined exclusively to vesicular structures colocalizing with a marker of the HLA class II loading compartment (HLA-DM). In contrast, gH was dispersed evenly throughout the cytoplasm, and no costaining with HLA-DM was observed.

It remains possible that the pattern of glycoprotein expression may be different in the context of viral infection, as the cytoplasmic viral assembly compartment is indeed located to the vesicular structures of infected cells (45, 69). However, budding virus colocalized with secretory vesicles more so than with late endosomes or lysosomes (endocytic vesilcles), suggesting that other glycoproteins may not be able to enter the HLA class II processing pathway very efficiently in vivo. This would result in a much more efficient presentation of gB-derived epitopes in the context of HLA class II from within virus-infected cells compared with other structural CMV proteins and therefore may offer an explanation for the substantially reduced immunodomiance of gH in comparison with gB.

The mechanism by which gB gains entry into the endosomal processing pathway has been proposed to be due to sorting sequences within the C-terminal domain of the protein (39). We deleted this domain from gB, in the gBΔCT construct, but found, somewhat to our surprise, that a gB-derived epitope could still be generated via an endogenous processing route from this protein, albeit at a slightly reduced level of efficiency. This demonstrates that the C-terminal domain is not the only mechanism that allows gB to enter into the endosomal pathway. This observation was confirmed by analysis of the pattern of cellular expression of the gBΔCT protein. Although this did demonstrate some degree of cytoplasmic expression, the deletion did not completely abrogate transport of gB into the HLA class II pathway.

Additionally, transfer of the C-terminal domain onto the gH protein did not lead to generation of the gH-derived epitope in an endogenous manner. Indeed, the gH-gBCT protein was not detected in any vesicular structures despite the attachment of the entire C terminus from gB to its C-terminal region. This indicates that factors within the gB and gH proteins themselves, possibly relating to secondary structures embedded within the remaining protein domains, for example the transmembrane domain (70), serve to contribute to the pattern of endosomal localization. Additionally, other mechanisms may come into play that allow intracellular generation of CD4^+^ T cell epitopes in a proteasome- and TAP-independent manner for presentation in MHC class II (71, 72). This will however need further investigation.

In summary, our results demonstrate the remarkable T cell immunodominance of gB in comparison with gH and gL in the number and range of epitopes contained within the protein and also differences in the nature of the functional response elicited by glycoprotein-specific T cells. This most likely reflects the fact that gB is the only protein that is directed into the endosomal HLA class II presentation pathway, a process partly, but not completely, mediated by sequences within the C-terminal domain. These observations on the unique properties of the gB-specific T cell response support its role as a core component within a HCMV vaccine. However, RhCMV and MCMV vaccine models have demonstrated the increased efficacy of multivalent vaccines, and in all cases induction of a cellular response correlated with improved outcome (73, 74). The next generation HCMV vaccines are also likely to incorporate additional viral proteins, and gH and gL, as part of the pentameric complex, are leading candidates in this regard. Our results indicate that future vaccine design strategies should incorporate approaches that ensure the generation of adequate cellular immunity against these glycoproteins.

## Supplementary Material

Data Supplement
