# Supplementary material for: The Cellular Localization of Human Cytomegalovirus Glycoprotein Expression Greatly Influences the Frequency and Functional Phenotype of Specific CD4+ T Cell Responses
Source: J Immunol. 2015 Sep 11;195(8):3803–15. doi: 10.4049/jimmunol.1500696 (PMC4592104; doi:10.4049/jimmunol.1500696)
Supplement: Data Supplement [file JI_1500696.zip › JI_1500696_Supplemental_Material_1.pdf]

pool	1	2	3	4	5	6	7	8	9	10	11	12	13
**14**	1	2	3	4	5	6	7	8	9	10	11	12	13
**15**	14	15	16	17	18	19	20	21	22	23	24	25	26
**16**	27	28	29	30	31	32	33	34	35	36	37	38	39
**17**	40	41	42	43	44	45	46	47	48	49	50	51	52
**18**	53	54	55	56	57	58	59	60	61	62	63	64	65
**19**	66	67	68	69	70	71	72	73	74	75	76	77	78
**20**	79	80	81	82	83	84	85	86	87	88	89	90	91
**21**	92	93	94	95	96	97	98	99	100	101	102	103	104
**22**	105	106	107	108	109	110	111	112	113	114	115	116	117
**23**	118	119	120	121	122	123	124	125	126	127	128	129	130
**24**	131	132	133	134	135	136	137	138	139	140	141	142	143
**25**	144	145	146	147	148	149	150	151	152	153	154	155	156
**26**	157	158	159	160	161	162	163	164	165	166	167	168	169
**27**	170	171	172	173	174	175	176	177	178	179			

Supplementary Figure 1. Glycoprotein B cross matrix of peptide pools designed for screening. Peptides 20 amino acids in length, overlapping by fifteen amino acids, spanning the whole protein sequence were generated. Twenty-seven peptide pools were made up according to the depicted scheme. The number of pools was determined by the square root of the total number of peptides. Each pool contained 10-14 peptides whereby each peptide is contained in exactly two of the pools. Fields shaded in grey highlight previously published epitopes.

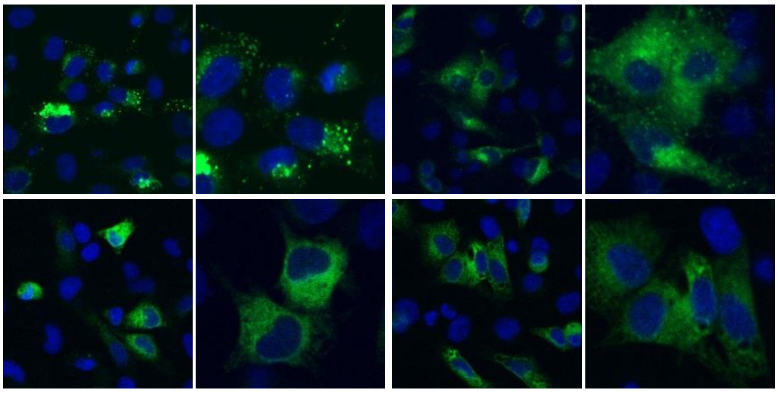

Supplementary Figure 2. The cellular distribution pattern of CMV glycoproteins differs greatly. MJS cells were transfected with GFP-expressing constructs of gB-WT, gBΔCT, gH-WT and gH-gBCT before fixation 24h later. They were counterstained with DAPI and mounted on microscope slides. gB-WT protein displayed a very distinct localisation exclusively to vesicular structures (top left) whereas gH-WT was evenly dispersed throughout the cytoplasm of the cell (bottom left). gBΔCT on the other hand showed both vesicular and cytoplasmic localisation (top right). Distribution of gH-gBCT closely resembled that of the wild type protein (bottom right).

Supplementary Table I. HLA class II types and age of donors in the study.

Donor	age [yrs]	HLA-DR				HLA-DQ		HLA-DP	
**1**	58	1	7		53	5		not known	
**2**	29		7		53	2	9	2	4
**3**	48	11	15	51	52b	6	7	not known	
**4**	27	7	17	52a	53	2	2	2	4
**5**	43	7	16	51	53	2	5	1	1
**6**	48	7	11	52b	53	7	9	4	11
**7**	44	4			53	8	-	not known	
**8**	52	1	13.01	52a		5	6	4	14
**9**	40	4	7		53	2	4	not known	
**10**	26	4	16	51	53	5	8	3	14
**11**	53	13	17	52a	52b	2	6	2	8
**12**	45	4	10		53	5	8	4	26
**13**	48	1	13.01	52b		5	6	not known	
**14**	68	7	17	52a	53	2	9	not known	
**15**	30	15	17	51	52b	2	6	4	-
**16**	49	4	15	51	53	6	7	4	5
**17**	40	1	16	51		5	5	4	9
**CMV-ve**	27	7	14	52b	53	2	5	4	-

Supplementary Table II. Summary of T-cell peptide epitopes identified in glycoprotein B, glycoprotein H and glycoprotein L.

Antigen	sequence	co-ordinates	restriction	response size % of CD4/8 T cells (range)	reference
**gB**	DLIRFERNIICTSMKPINED	101-120	CD4	0.04-0.041	
	CTSMKPINEDLDEGIMVVYK	111-130	CD4	0.24	
	MVVYKRNIVAHTFKVRVYQK	126-145	CD4	0.24	
	RSYAYIYTTYLLGSNTEYVA	151-170	CD4	0.02-1.04	
	TEYVAPPMWEIHHINKFAQC	166-185	CD4	0.024	
	IHH**INKFAQCYSSYSRVIG**G	176-195	CD4	0.024-0.034	(32)
	YHRDSYENKTMQLIPDDYSN	201-220	CD4	0.898	
	MQLIPD**DYSNTHSTRYV**TVK	211-230	CD4	0.03-1.59	(37)
	**MLTITTARSKYPYH**FFATST	251-270	CD4	0.09	(32)
	FATSTGDVVYISPFYNGTNR	266-285	CD4	0.019	
	GDVVYISPFYNGTNRNASYF	271-290	CD4	0.012	
	NGTNRNASYFGENADKFFIF	281-300	CD4	0.021	
	NASYFGENADKFFIFPNYTI	286-305	CD4	0.7	
	PNYTIVSDFGRPNAAPETHR	301-320	CD4	0.02	
	LTFWEASERTIRSEAEDSYH	346-365	CD4	0.23	
	IRSEAEDSYHFSSAKMTATF	356-375	CD4	0.04-0.44	
	FSSAKMTATFLSKKQEVNMS	366-385	CD4	0.009-0.32	
	DSALDCVRDEAINKLQQIFN	386-405	CD4	0.05	
	GNVS**VFETSGGLVVFWQGI**K	416-435	CD4	0.02-0.15	(32, 36)
	FETSGGLVVFWQGIKQKSLV	421-440	CD4	0.05-0.64	
	TYDTLRGYINRALAQIAEAW	486-505	CD4	0.17	
	RTLEVFKELSKINPSAILSA	511-530	CD4	0.31	
	MGDVLGLASCVTINQTSVKV	541-560	CD4	0.17	
	RTEECQLPSLKIFIAGNSAY	606-625	CD4	0.019	
	KIFIAGNSA**YEYVDYLFKRM**	616-635	CD4	0.019	(36)
	PLQNLFPYLVSADGTTVTSG	781-800	CD4	0.16	
	FPYLVSADGTTVTSGSTKDT	786-805	CD4	0.69	
	NEQAYQMLLALARLDAEQRA	841-860	CD4	0.01-1.8	
	DSLDGQTGTQDKGQKPNLLD	866-885	CD4	0.17	
	MVVYKRNIVAHTFKVRVYQK	126-145	CD8	0.15	
	VLTFRRSYAYIYTTYLLGSN	146-165	CD8	0.27	
**gH**	VFTVYLLSHLPSQRYGADAA	11-30	CD4	0.008	
	TYNSSLRNSTVVRENAISFN	76-95	CD4	0.24	
	AISFNFFQSYNQYYVFHMPR	61-80	CD4	0.017	
	DLTETLERYQQRLNTYALVS	111-130	CD4	0.35	
	QRLNTYALVSKDLASYRSFS	121-140	CD4	0.35	
	SHTTSGLHRPHFNQTCILFD	181-200	CD4	0.14	
	QLNRHSYLKDSDFLDAALDF	286-305	CD4	1.14	
**gL**	QGDKYESWLRPLVNVTRRDG	61-80	CD4	0.032	
	RTNRAVRLPVSTAAAPEGIT	201-220	CD4	0.02	
	VRLPVSTAAAPEGITLFYGL	206-225	CD8	0.014	

Previously published epitopes are highlighted in bold
Where a T-cell response was detected in more than one donor, the range of the response size is shown.
